# Fentanyl activates opposing opioid and non-opioid receptor systems that control breathing

**DOI:** 10.3389/fphar.2024.1381073

**Published:** 2024-04-18

**Authors:** Santhosh M. Baby, Walter J. May, Paulina M. Getsy, Gregory A. Coffee, Tej Nakashe, James N. Bates, Alan Levine, Stephen J. Lewis

**Affiliations:** ^1^ Department of Drug Discovery, Galleon Pharmaceuticals, Inc., Horsham, PA, United States; ^2^ Pediatric Respiratory Medicine, University of Virginia School of Medicine, Charlottesville, VA, United States; ^3^ Department of Pediatrics, Case Western Reserve University, Cleveland, OH, United States; ^4^ Department of Biological Sciences, Kent State University, Kent, OH, United States; ^5^ Department of Anesthesiology, University of Iowa Hospitals and Clinics, Iowa City, IO, United States; ^6^ Department of Molecular Biology and Microbiology, Case Western Reserve University, Cleveland, OH, United States; ^7^ Department of Pharmacology, Case Western Reserve University, Cleveland, OH, United States

**Keywords:** fentanyl, breathing, opioid receptors, naloxone methiodide, rats

## Abstract

Fentanyl elicits profound disturbances in ventilatory control processes in humans and experimental animals. The traditional viewpoint with respect to fentanyl-induced respiratory depression is that once the effects on the frequency of breathing (Freq), tidal volume (TV), and minute ventilation (MV = Freq × TV) are resolved, then depression of breathing is no longer a concern. The results of the present study challenge this concept with findings, as they reveal that while the apparent inhibitory effects of fentanyl (75 μg/kg, IV) on Freq, TV, and MV in adult male rats were fully resolved within 15 min, many other fentanyl-induced responses were in full effect, including opposing effects on respiratory timing parameters. For example, although the effects on Freq were resolved at 15 min, inspiratory duration (Ti) and end inspiratory pause (EIP) were elevated, whereas expiratory duration (Te) and end expiratory pause (EEP) were diminished. Since the effects of fentanyl on TV had subsided fully at 15 min, it would be expected that the administration of an opioid receptor (OR) antagonist would have minimal effects if the effects of fentanyl on this and other parameters had resolved. We now report that the intravenous injection of a 1.0 mg/kg dose of the peripherally restricted OR antagonist, methyl-naloxone (naloxone methiodide, NLXmi), did not elicit arousal but elicited some relatively minor changes in Freq, TV, MV, Te, and EEP but pronounced changes in Ti and EIP. In contrast, the injection of a 2.5 mg/kg dose of NLXmi elicited pronounced arousal and dramatic changes in many variables, including Freq, TV, and MV, which were not associated with increases in non-apneic breathing events such as apneas. The two compelling conclusions from this study are as follows: 1) the blockade of central ORs produced by the 2.5 mg/kg dose of NLXmi elicits pronounced increases in Freq, TV, and MV in rats in which the effects of fentanyl had apparently resolved, and 2) it is apparent that fentanyl had induced the activation of two systems with counter-balancing effects on Freq and TV: one being an opioid receptor inhibitory system and the other being a non-OR excitatory system.

## Introduction

The high-potency μ-opioid receptor agonist, fentanyl, is an important clinical analgesic that has adverse side effects, including ventilatory depression and the development of hyperalgesia (enhanced pain perception) upon repeated administration (Scholz et al., 1996; Suzuki and El-Haddad, 2017; Armenian et al., 2018; Tyler and Guarnieri, 2023). In addition, although the systemic injection of fentanyl causes profound analgesia in rats, it also exerts adverse effects on ventilatory parameters, the alveolar–arterial (A-a) gradient (index of alveolar gas exchange), and arterial blood–gas chemistry (Henderson et al., 2014; Baby et al., 2021; Jenkins et al., 2021; Getsy et al., 2022a; Getsy et al., 2022b; Seckler et al., 2022; Marchette et al., 2023). The roles of μ-, δ-, and κ-opioid receptors (Trescott et al., 2008; Valentino and Volkow, 2018), cell-signaling/molecular mechanisms (Al-Hasani and Bruchas, 2011; Shang and Filizola, 2015), and multiple sites of action of opioids (May et al., 1989; Lalley, 2003; Vadivelu et al., 2011; Henderson et al., 2013; 2014; Baby et al., 2018; Birdsong and Williams, 2020; Rossi and Bodnar, 2021) have been extensively studied. With regard to *in vivo* pharmacological approaches, the acute systemic administration of centrally acting opioid receptor antagonists such as naloxone and peripherally restricted antagonists such as methyl-naloxone (naloxone methiodide, NLXmi) has provided important evidence pertaining to the central and peripheral sites involved in mediating the actions of opioids (Lewanowitsch and Irvine, 2002; 2003; Lewanowitsch et al., 2006; Leppert, 2010; Yamamoto and Sugimoto, 2010; Mori et al., 2013; Henderson et al., 2014; Belltall et al., 2022).

We have reported that the ventilatory responses that occur during exposure to hypoxic or hypoxic–hypercapnic gas challenges were markedly attenuated in freely-moving adult male rats in which baseline ventilatory parameters had recovered from the adverse effects of morphine injected intravenously 90 min earlier (May et al., 2013a,b). These findings suggested that although baseline ventilation returned to normal values, the cell-signaling pathways activated by morphine or its major metabolites, including morphine-3 glucuronide in rats (Ekblom et al., 1993; Baker and Ratka, 2002; Blomqvist et al., 2020), are still active and prevented full recruitment and activation of central and peripheral pathways that mediate the ventilatory responses to these gas challenges (May et al., 2013a,b; Baby et al., 2023). Moreover, while the systemic administration of morphine-6-glucuronide, the major morphine metabolite in humans (Hanna et al., 1991; Peat et al., 1991; Christrup, 1997), did not alter baseline ventilation in healthy human volunteers, it strongly suppressed ventilatory responses to a hypercapnic gas challenge (Peat et al., 1991). The question that arises is what sort of latent ventilatory control system is present and active without disturbing baseline ventilation but is able to blunt ventilatory responses to hypoxic and hypercapnic challenges? One hypothesis that came to mind was that opioids such as morphine and fentanyl and/or their metabolites may elicit counter-balancing excitatory and inhibitory systems in the brain (and perhaps peripheral structures such as the carotid bodies) such that baseline ventilatory parameters appear normal. It would follow that selectively disturbing one of these excitatory or inhibitory systems at a time when baseline ventilatory parameters appear to have recovered from the opioid will elicit pronounced changes in ventilation. We hypothesize that the inhibitory system is driven by μ-opioid receptors and that the injection of an opioid receptor antagonist will elicit a pronounced ventilatory response.

The major goal of this study was to determine whether the putative excitatory ventilatory system activated by fentanyl resides within the periphery and/or central nervous system. To this end, we employed a dose of NLXmi (1.0 mg/kg, IV) that our evidence suggests does not enter the brain in sufficient amounts to effectively block μ-opioid receptors and a higher dose (2.5 mg/kg, IV) that does. The major experimental objectives were to 1) inject a dose of fentanyl (75 μg/kg, IV) that causes prompt sedation (loss of righting reflex) and substantial changes in ventilatory parameters in male Sprague Dawley rats, 2) determine when frequency of breathing (Freq), tidal volume (TV), and minute ventilation (MV) had fully recovered (as the generally accepted parameters describing the effects of drugs on ventilation, and 3) at this time, inject a dose of NLXmi (1.0 mg/kg, IV) that does not arouse these fentanyl-injected rats (suggestive of restriction to the periphery) or a dose of NLXmi (2.5 mg/kg, IV) that causes a pronounced arousal (restoration of the righting reflex suggestive of significant central penetration).

## Methods

### Permissions, rats, and surgical procedures

All studies were carried out in accordance with the NIH Guide for Care and Use of Laboratory Animals (NIH Publication No. 80-23) revised in 2011 and in compliance with the ARRIVE (Animal Research: Reporting of *In Vivo* Experiments) guidelines (https://arriveguidelines.org/). All protocols involving rats were approved by the Animal Care and Use Committees of Galleon Pharmaceuticals, Case Western Reserve University, and the University of Virginia. Adult male and female Sprague Dawley rats (10–12 weeks of age) were purchased from *Harlan Industries* (Madison, WI, United States) and were given 5 days to recover from transportation. The rats received jugular vein catheters under 2%–3% isoflurane anesthesia (May et al., 2013a; May et al., 2013b; Henderson et al., 2013; Henderson et al., 2014) and given 4 days to recover from surgery before use. All venous catheters were flushed with 0.3 mL of phosphate-buffered saline (0.1 M, pH 7.4) 3–4 h before starting the protocols. Plethysmography recording sessions were performed by an investigator who did not know whether they were injecting a vehicle or the 1.0 or 2.5 mg/kg doses of NLXmi. Fentanyl citrate powder and naloxone methiodide (NLXmi, N-methylnaloxonium iodide) powder (product number: N12P; PubChem Substance ID: 24897480; CAS number: 93302-47-7) were purchased from *Sigma-Aldrich* (St. Louis, MO, United States) and dissolved in normal saline just before use. Data files resulting from each study were collated and analyzed by another investigator in the group. Note that each rat was involved in only one protocol and was not re-used in any other study. Figures and diagrams describing the plethysmography set-up are found at the *Data Sciences International* site at https://www.datasci.com/products/buxco-respiratory-products. All studies were conducted in a quiet room with a relative humidity of 49% ± 2% and a temperature of 21.4°C ± 0.2 C.

### Sedation as determined by the modified righting reflex test

This study evaluated the effects of injections of vehicle and NLXmi on the duration of the effects of fentanyl on the modified righting reflex test. Each rat was placed in an open container to evaluate the loss of reflex. The administration of fentanyl caused the rats to assume numerous types of postures, including being motionless, sprawled out on their stomach on the chamber floor, lying motionless on their side, and splayed out on their stomach with the head up against the chamber wall. The duration of the fentanyl sedation was taken as the time to full recovery of the righting reflex (i.e., when rats attained and maintained a normal posture on all four legs) following the injections of vehicle or NLXmi (Gaston et al., 2021; Getsy et al., 2022c; Getsy et al., 2022d; Getsy et al., 2022e; Lewis et al., 2022). **Male rats:** one group (304 ± 2 g in body weight, n = 9) received an injection of fentanyl (75 μg/kg, IV), followed by an injection of vehicle (saline) after 15 min. A second group (302 ± 2 g in body weight, n = 9) received fentanyl (75 μg/kg, IV), followed by an injection of NLXmi (1.0 mg/kg, IV) after 15 min. A third group (303 ± 2 g in body weight, n = 9) received fentanyl (75 μg/kg, IV), followed an injection of NLXmi (2.5 mg/kg, IV) after 15 min. **Female rats:** one group (279 ± 2 g in body weight, n = 9) received an injection of fentanyl (75 μg/kg, IV), followed by an injection of vehicle (saline) after 15 min. A second group (281 ± 2 g in body weight, n = 9) received fentanyl (75 μg/kg, IV), followed by an injection of NLXmi (1.0 mg/kg, IV) after 15 min. A third group (278 ± 2 g in body weight, n = 9) received fentanyl (75 μg/kg, IV), followed by an injection of NLXmi (2.5 mg/kg, IV) after 15 min.

### Protocols for whole-body plethysmography measurement of ventilatory parameters

Ventilatory parameters were recorded continuously in the unrestrained, freely-moving rats using a whole-body plethysmography system (PLY3223; *Data Sciences International*, St. Paul, MN), as detailed previously (Jenkins et al., 2021; Getsy et al., 2022a; Getsy et al., 2022b; Seckler et al., 2022; Seckler et al., 2023). The directly recorded and derived parameters are defined in Supplemental Table S1. The ventilatory parameters and abbreviations are as follows: frequency of breathing (Freq), tidal volume (TV), minute ventilation (MV), inspiratory time (Ti), expiratory time (Te), Ti/Te, end inspiratory pause (EIP), end expiratory pause (EEP), peak inspiratory flow (PIF), peak expiratory flow (PEF), PIF/PEF, expiratory flow at 50% expired TV (EF_50_), relaxation time (RT), expiratory delay (Te-RT), inspiratory drive (InspD, TV/Ti), expiratory drive (ExpD, TV/Te), non-eupneic breathing index (NEBI, % of non-eupneic breathing events per each epoch), and NEBI corrected for Freq (NEBI/Freq). A diagram adapted from Lomask (2006) illustrating the relationships between some recorded parameters is shown in Supplemental Figure S1. On the day of study, each rat was placed in a plethysmography chamber and allowed 60 min to acclimatize for resting (baseline, pre) ventilatory parameter values to be defined. Two sets of studies were conducted. **Study 1:** Three groups of rats (n = 6 per group) received an injection of vehicle (1.0 mL/kg, IV), and after 15 min, one group (304 ± 3 g in body weight) received an injection of vehicle (saline), a second group (302 ± 3 g) received an injection of NLXmi (1.0 mg/kg, IV), and a third group (305 ± 3 g) received an injection of the higher dose of NLXmi (2.5 mg/kg, IV). **Study 2:** Three groups of rats (n = 4 per group) received an injection of fentanyl (75 μg/kg, IV), and after 15 min, one group (301 ± 2 g in body weight) was injected with vehicle (saline), a second group (299 ± 2 g) was injected with NLXmi (1.0 mg/kg, IV), and a third group (302 ± 2 g) was injected with the higher dose of NLXmi (2.5 mg/kg, IV). **Study 3:** Three groups of female rats (n = 4 per group) received an injection of fentanyl (75 μg/kg, IV), and after 15 min, one group (280 ± 2 g in body weight) was injected with vehicle (saline), a second group (279 ± 2 g) was injected with NLXmi (1.0 mg/kg, IV), and a third group (277 ± 2 g in body weight) was injected with the higher dose of NLXmi (2.5 mg/kg, IV). Ventilatory parameters were recorded for another 60 min. *FinePointe* (DSI) software constantly corrected digitized values originating from actual waveforms for alterations in chamber humidity and chamber temperature. Pressure changes associated with respiratory waveforms were then converted to volumes (e.g., TV, PIF, PEF, and EF_50_) using the algorithms of Epstein and Epstein (1978) and Epstein et al. (1980). Factoring in chamber humidity and temperature, cycle analyzers filtered the acquired signals, and *FinePointe* algorithms generated an array of box flow data that identified a waveform segment as an acceptable breath. From that data vector, the minimum and maximum values were determined. Flows at this point were “box flow” signals, and from this array, the minimum and maximum box flow values were multiplied by a compensation factor provided by the selected algorithm (Epstein and Epstein, 1978; Epstein et al., 1980), producing TV, PIF, and PEF values used to determine NEBI (Getsy et al., 2014).

### Data analyses

All data are presented as the mean ± SEM and were analyzed using one-way and two-way ANOVA and Bonferroni corrections for multiple comparisons between means using the error mean square terms generated using the ANOVA analyses (Getsy et al., 2023a; Getsy et al., 2023b). A *p* < 0.05 value was the initial level of significance that was modified according to the number of between-mean comparisons (Getsy et al., 2023a; Getsy et al., 2023b). The modified *t-*statistic for two groups, for instance, is t = (mean group 1 ˗ mean group 2)/[s × (1/n_1_ + 1/n_2_)^1/2^], where s^2^ represents the mean square within groups term from the ANOVA and n_1_ and n_2_ are the number of rats in each group being compared. Statistics were performed using GraphPad Prism (version 9.5.1) software (*GraphPad Software*, Inc., La Jolla, CA). F- and *P*-statistics related to the summary data are provided in the relevant figure legends and tables.

## Results

### Behavioral responses in male and female rats

**FIGURE 1:**
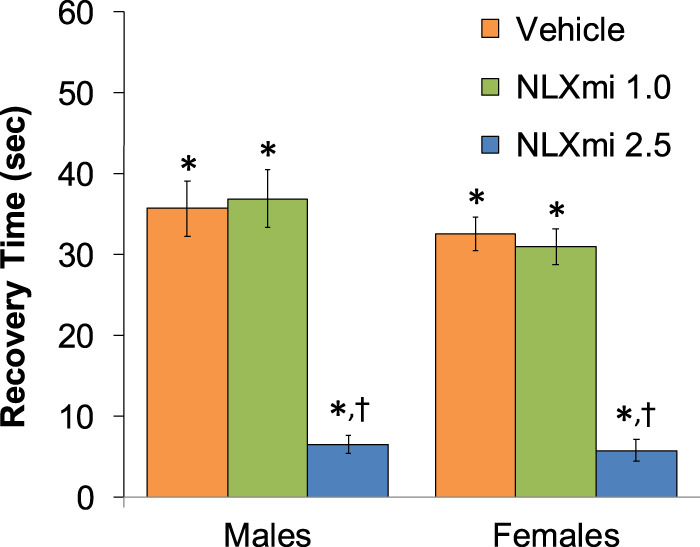
Time to recovery of the righting reflex following the bolus injection of vehicle (saline) and subsequent injection of vehicle or NLXmi (1.0 or 2.5 mg/kg, IV) in male and female rats. There were nine rats in each group. The data are presented as the mean ± SEM. *p < 0.05, significant Pre-values. ^†^ *p* < 0.05, NLXmi 2.5 versus NLXmi 1.0 or vehicle. **ANOVA statistics for males:** F_2,24_ = 34.5; *p* < 0.0001. **ANOVA statistics for females:** F_2,24_ = 48.5; *p* < 0.0001.

The injection of fentanyl (75 μg/kg, IV) elicited prompt sedation (loss of righting reflex) in the three groups of male rats and the three groups of female rats. As shown in Figure 1, the time to recovery of the righting reflex following the subsequent injection of vehicle or 1.0 mg/kg dose of NLXmi was similar to one another (*p* > 0.05) in the male and female rats. In contrast, the duration of the righting time in the 2.5 mg/kg NLXmi-treated male and female rats was substantially shorter than that in the other two respective treatment groups (*p* < 0.05 for both comparisons.

### Frequency of breathing, tidal volume, and minute ventilation in male rats

**FIGURE 2:**
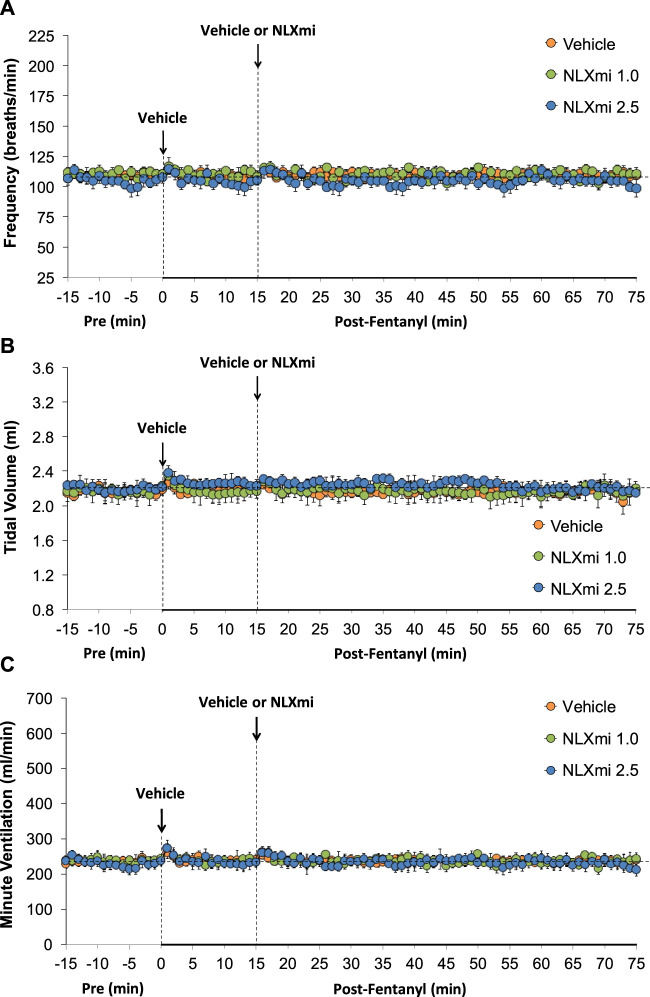
Frequency of breathing **(Panel A)**, tidal volume **(Panel B)**, and minute ventilation **(Panel C)** before (Pre) and after the injection of vehicle (saline) and subsequent injection of vehicle or NLXmi (1.0 or 2.5 mg/kg, IV) in male rats. There were six rats in each group. The data are presented as the mean ± SEM.

**FIGURE 3:**
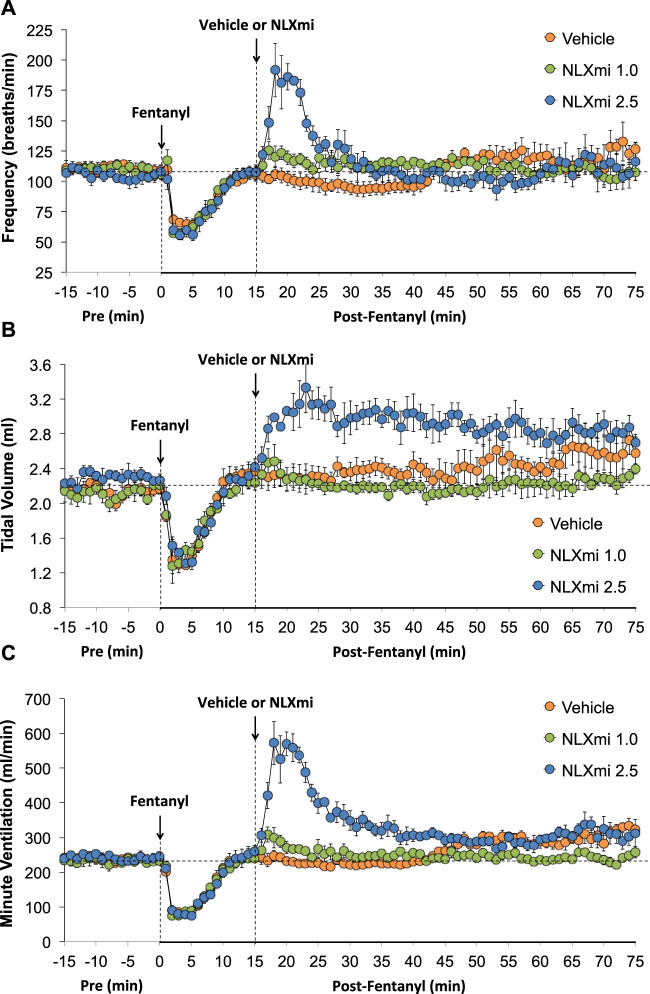
Frequency of breathing **(Panel A)**, tidal volume **(Panel B)**, and minute ventilation **(Panel C)** before (Pre) and after the injection of fentanyl (75 μg/kg, IV) and subsequent injection of vehicle or NLXmi (1.0 or 2.5 mg/kg, IV) in male rats. There were four rats in each group. The data are presented as the mean ± SEM.

As shown in Figure 2, the injections of vehicle or NLXmi (1.0 mg/kg, IV) elicited minor changes in Freq (**Panel A**), TV (**Panel B**), and MV (**Panel C**) in rats that had received injections of vehicle (no behavioral responses resulted from the NLXmi injections). As shown in Figure 3, the injection of fentanyl (75 μg/kg, IV) elicited pronounced decreases in Freq (**Panel A**), TV (**Panel B**), and MV (**Panel C**) that had fully resolved within 10–12 min. The subsequent injection of vehicle or NLXmi (1.0 mg/kg, IV) at 15 min post-fentanyl did not elicit immediate responses, whereas the injection of NLXmi at 2.5 mg/kg elicited a prompt increase in Freq of approximately 10 min in duration and a prompt and sustained increase in TV. The changes in Freq and TV resulted in a prompt increase in MV of approximately 25–30 min in duration. Note that the minor degree of variability in baseline (pre-fentanyl) levels of Freq and TV that resulted in a consistent level of MV is to be expected when recording values from freely-moving rats (Henderson et al., 2013; 2014; Gaston et al., 2020; Getsy et al., 2014; Gaston et al., 2021; Jenkins et al., 2021; Getsy et al., 2022a; Getsy et al., 2022b; Getsy et al., 2022c; Getsy et al., 2022d; Getsy et al., 2022e; Lewis et al., 2022; Seckler et al., 2022).

### Inspiratory time, expiratory time, Ti/Te, EIP, and EEP in male rats

**FIGURE 4:**
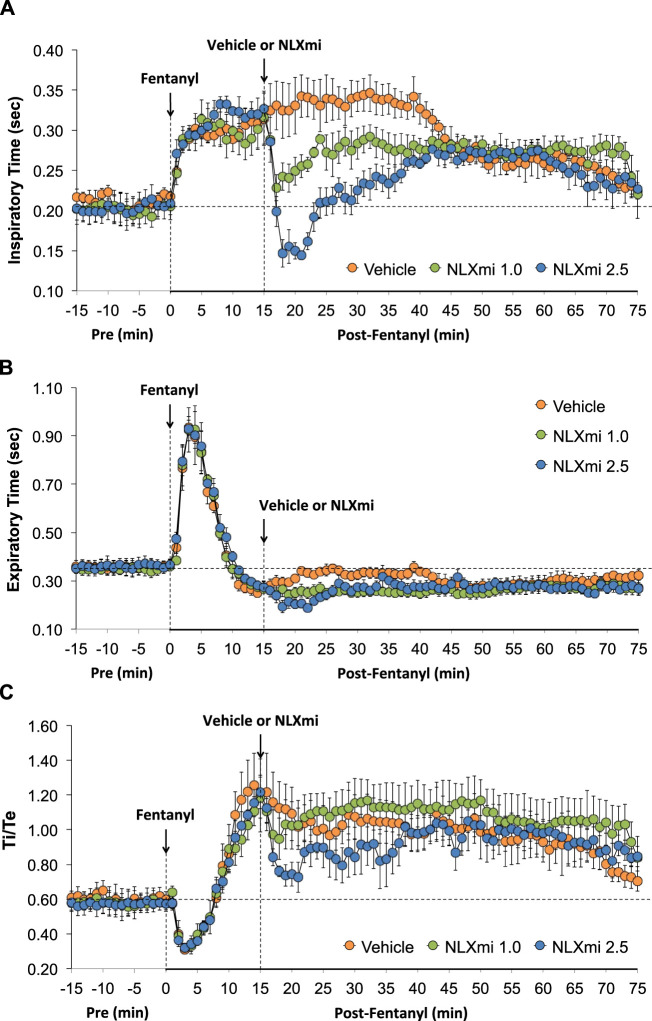
Inspiratory time (Ti) **(Panel A)**, expiratory time (Te) **(Panel B)**, and Ti/Te **(Panel C)** before (Pre) and after the injection of fentanyl (75 μg/kg, IV) and subsequent injection of vehicle or NLXmi (1.0 or 2.5 mg/kg, IV) in male rats. There were four rats in each group. The data are presented as the mean ± SEM.

As shown in Figure 4, the injection of fentanyl elicited an immediate and sustained increase in Ti (**Panel A**) and a prompt increase in Te of less than 10 min in duration (**Panel B**). As shown in **Panel C**, these changes resulted in a biphasic change in the respiratory ratio (Ti/Te, initial decrease and then a sustained increase). The subsequent injection of vehicle did not elicit immediate responses, whereas the injection of NLXmi at 1.0 or 2.5 mg/kg elicited prompt decreases in Ti and Te of approximately 20 min in duration, with the elevated Ti/Te ratios slightly more decreased by the 2.5 mg/kg dose.

### End inspiratory pause and end expiratory pause in male rats

**FIGURE 5:**
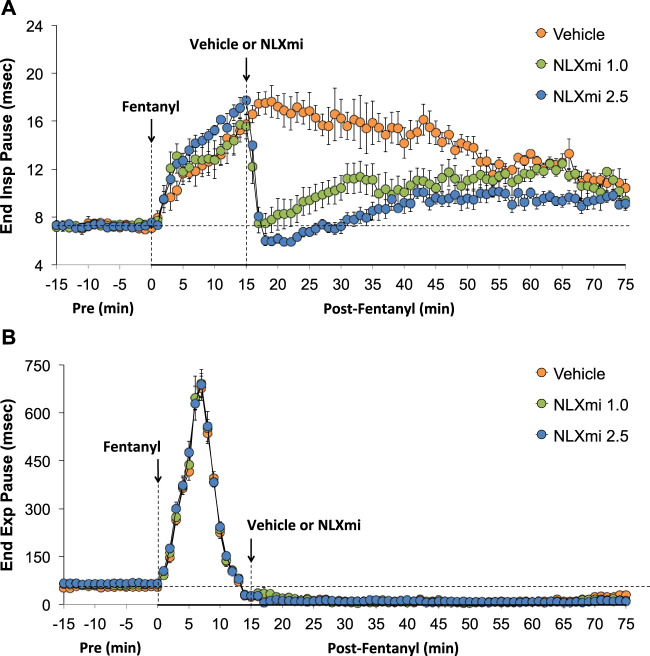
End inspiratory pause **(Panel A)** and end expiratory pause **(Panel B)** before (Pre) and after the injection of fentanyl (75 μg/kg, IV) and subsequent injection of vehicle or NLXmi (1.0 or 2.5 mg/kg, IV) in male rats. There were four rats in each group. The data are presented as the mean ± SEM.

As shown in Figure 5, fentanyl elicited a sustained increase in EIP (**Panel A**) and a prompt increase in EEP that had dipped below pre-levels by 15 min (**Panel B**). The subsequent injection of vehicle did not elicit immediate responses, whereas the injection of NLXmi at 1.0 or 2.5 mg/kg elicited prompt decreases in the elevated levels of EIP for 35–40 min. Neither dose of NLXmi affected EEP, which remained depressed throughout the post-fentanyl recording period.

### Peak inspiratory flow, peak expiratory flow, PIF/PEF, and EF_50_ in male rats

**FIGURE 6:**
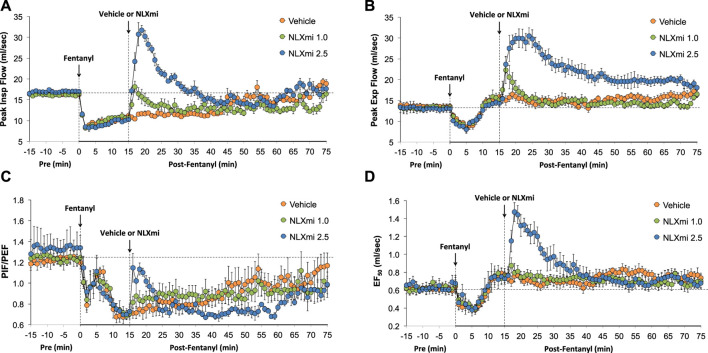
Peak inspiratory flow (PIF) **(Panel A)**, peak expiratory flow (PEF) **(Panel B)**, PIF/PEF **(Panel C)**, and expiratory flow at 50% expired tidal volume (EF_50_) **(Panel D)** before (Pre) and after the injection of fentanyl (75 μg/kg, IV) and subsequent injection of vehicle or NLXmi (1.0 or 2.5 mg/kg, IV) in male rats. There were four rats in each group. The data are presented as the mean ± SEM.

As shown in Figure 6, fentanyl elicited a sustained decrease in PIF (**Panel A**), a decrease in PEF of 6–8 min in duration (**Panel B**), a sustained decrease in PIF/PEF (**Panel C**), and a decrease in EF_50_ (**Panel D**) that returned to above pre-fentanyl levels after 10–12 min. The subsequent injection of NLXmi (1.0 mg/kg, IV) elicited relatively minor and short-lived increases in PIF, PEF, and PIF/PEF but not EF_50_. The injection of the 2.5 mg/kg dose of NLXmi elicited an immediate and substantial increase in PIF of 15–20 min in duration, a large and sustained increase in PEF of over 60 min in duration, an increase in PIF/PEF for approximately 5 min, and a substantial increase in EF_50_ of approximately 20 min in duration.

### Relaxation time and expiratory time–relaxation time in male rats

**FIGURE 7:**
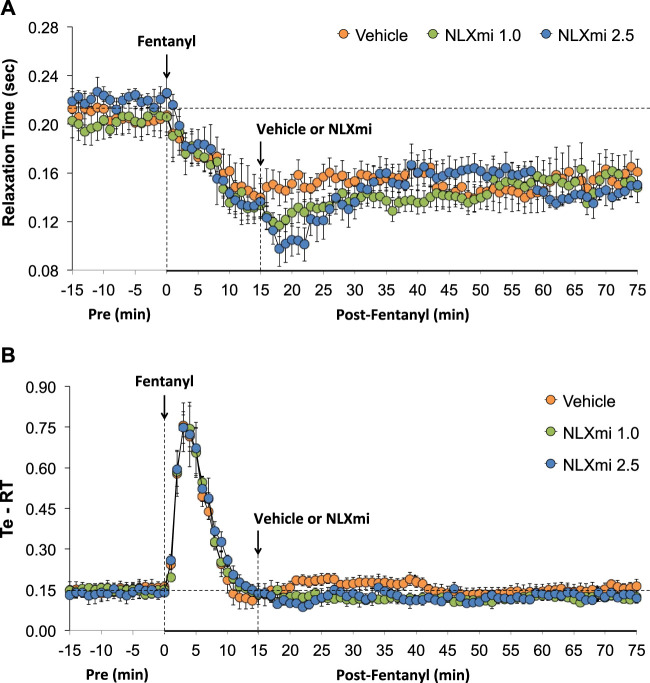
Relaxation time **(Panel A)** and expiratory time–relaxation Time (Te-RT) **(Panel B)** before (Pre) and after the injection of fentanyl (75 μg/kg, IV) and subsequent injection of vehicle or NLXmi (1.0 or 2.5 mg/kg, IV) in male rats. There were four rats in each group. The data are presented as the mean ± SEM.

As shown in Figure 7, fentanyl elicited a sustained decrease in RT (**Panel A**) and a substantial increase in Te-RT (expiratory delay) of approximately 10 min in duration (**Panel B**). The injection of 1.0 and 2.5 mg/kg doses of NLXmi elicited minor and short-lived decreases in RT and minor changes in expiratory time–relaxation time (Te-RT).

### Inspiratory drive and expiratory drive in male rats

**FIGURE 8:**
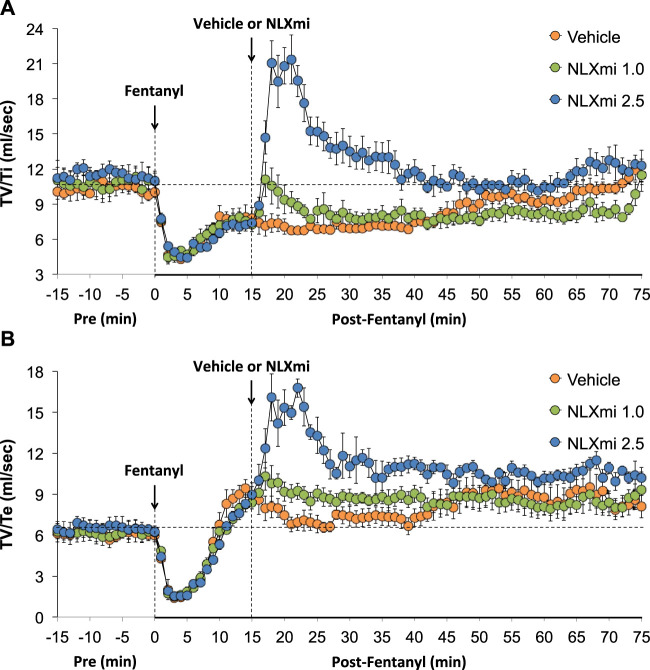
Inspiratory drive (tidal volume/inspiratory time, TV/Ti) **(Panel A)** and expiratory drive (tidal volume/expiratory time, TV/Te) **(Panel B)** before (Pre) and after the injection of fentanyl (75 μg/kg, IV) and subsequent injection of vehicle or NLXmi (1.0 or 2.5 mg/kg, IV) in male rats. There were four rats in each group. The data are presented as the mean ± SEM.

As shown in Figure 8, fentanyl elicited a sustained decrease in inspiratory drive (TV/Ti) (**Panel A**) and a substantial decrease in expiratory drive (TV/Te) (**Panel B**) of approximately 10 min in duration, with the levels at 15 min being above pre-injection levels. The subsequent injection of the 1.0 mg/kg dose of NLXmi elicited minor and short-lived decreases in TV/Ti and TV/Te. The subsequent injection of the 2.5 mg/kg dose of NLXmi elicited pronounced increases in TV/Ti and TV/Te of approximately 30 min in duration.

### Non-eupneic breathing index and NEBI/Freq in male rats

**FIGURE 9:**
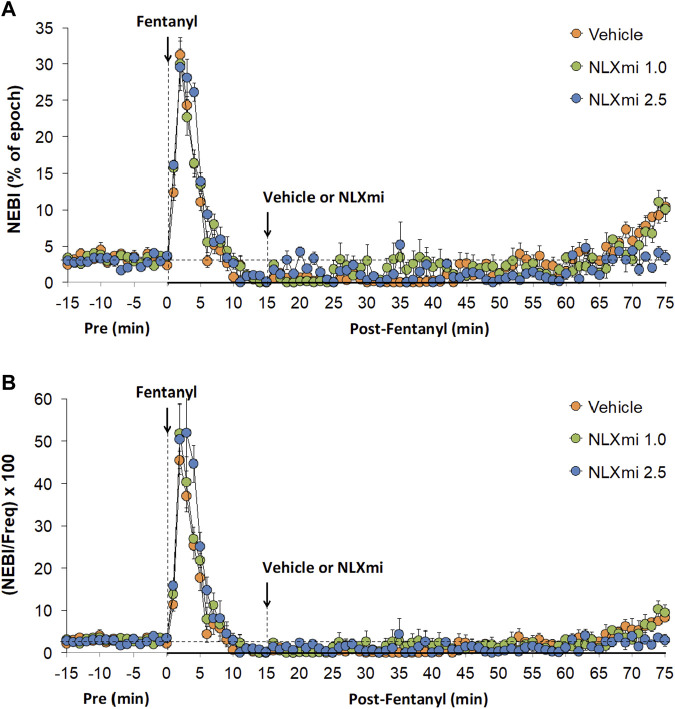
Non-eupneic breathing index **(Panel A)** and NEBI/frequency of breathing **(Panel B)** before (Pre) and after the injection of fentanyl (75 μg/kg, IV) and subsequent injection of vehicle or NLXmi (1.0 or 2.5 mg/kg, IV) in male rats. There were four rats in each group. The data are presented as the mean ± SEM.

As shown in Figure 9, fentanyl elicited substantial increases in NEBI (**Panel A**) and NEBI/Freq (**Panel B**) of approximately 5 min in duration. Subsequent injections of the 1.0 or 2.5 mg/kg doses of NLXmi elicited negligible changes in NEBI or NEBI/Freq.

### Summary of the effects of fentanyl on ventilation in male rats

**FIGURE 10:**
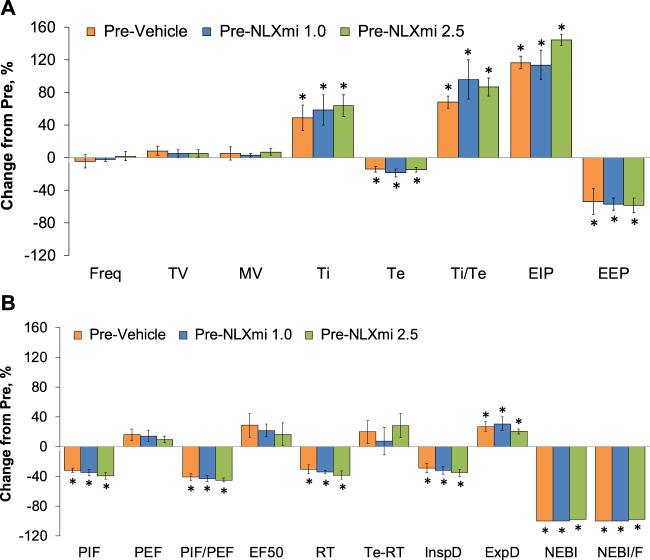
Ventilatory parameters (expressed as % change from Pre-values) [panels **(A,B)**] at the 15-min time-point following the injection of fentanyl (75 μg/kg, IV) in male rats that subsequently received an injection of vehicle or NLXmi (1.0 or 2.5 mg/kg, IV). There were four rats in each group. The data are presented as the mean ± SEM. *p < 0.05, significant change from Pre-values. There were no between-group differences for any parameter (*p* > 0.05 for all comparisons). **ANOVA statistics:** Freq: F_(2,9)_ = 0.29 and *p* = 0.756; TV: F_(2,9)_ = 0.49 and *p* = 0.628; MV: F_(2,9)_ = 0.14 and *p* = 0.875; Ti: F_(2,9)_ = 0.21 and *p* = 0.817; Te: F_(2,9)_ = 0.01 and *p* = 0.989; Ti/Te: F_(2,9)_ = 0.12 and *p* = 0.890; EIP: F_(2,9)_ = 4.68 and *p* = 0.040; EEP: F_(2,9)_ = 0.05 and *p* = 0.953; PIF: F_(2,9)_ = 0.79 and *p* = 0.484; PEF: F_(2,9)_ = 0.23 and *p* = 0.796; PIF/PEF: F_(2,9)_ = 0.28 and *p* = 0.760; EF_50_: F_(2,9)_ = 0.20 and *p* = 0.827; RT: F_(2,9)_ = 5.18 and *p* = 0.032; Te-RT: F_(2,9)_ = 0.07 and *p* = 0.935; InspD: F_(2,9)_ = 0.65 and *p* = 0.546; ExpD: F_(2,9)_ = 0.16 and *p* = 0.852; NEBI: F_(2,9)_ = 2.56 and *p* = 0.132; NEBI/F: F_(2,9)_ = 2.12 and *p* = 0.177.

The values for each recorded/calculated respiratory parameter (expressed as % change from pre) at the 15-min time-point following the injection of fentanyl (75 μg/kg, IV) in the three treatment groups are summarized in Figure 10. As can be seen in **Panel A**, the decreases in Freq, TV, and MV elicited by fentanyl had fully subsided at the 15-min time-point. However, the apparent lack of effect of fentanyl on Freq at the 15-min time-point was certainly misleading in that Ti and EIP were elevated, whereas Te and EEP were decreased at this time-point. Accordingly, it is evident that the effects of fentanyl (or metabolites) on inspiratory–expiratory timing mechanisms are still in effect. Moreover, as shown in **Panel B**, the fentanyl-induced changes in PIF (but not PEF or EF_50_), relaxation time, NEBI, and NEBI/Freq were still in effect with the decreases in inspiratory drive (TV/Ti) and increases in expiratory drive (ExpD, TV/Te), resulting mostly from respective changes in Ti and Te. The cumulative responses (% change from pre-values) recorded over the 15-min period following the injection of fentanyl (75 μg/kg, IV) in rats that were to receive subsequent injections of vehicle or NLXmi (1.0 or 2.5 mg/kg, IV) are summarized in Supplemental Figure S3. The values reinforce the above descriptions of the effects of fentanyl, noting pronounced decreases in Freq, TV, MV, RT, PIF, PIF/PEF, inspiratory drive, and expiratory drive that were associated with pronounced increases in Ti, Te, EIP, EEP, Ti-RT, NEBI, and NEBI/Freq.

### Summary of the effects of NLXmi on ventilation in male rats

**FIGURE 11:**
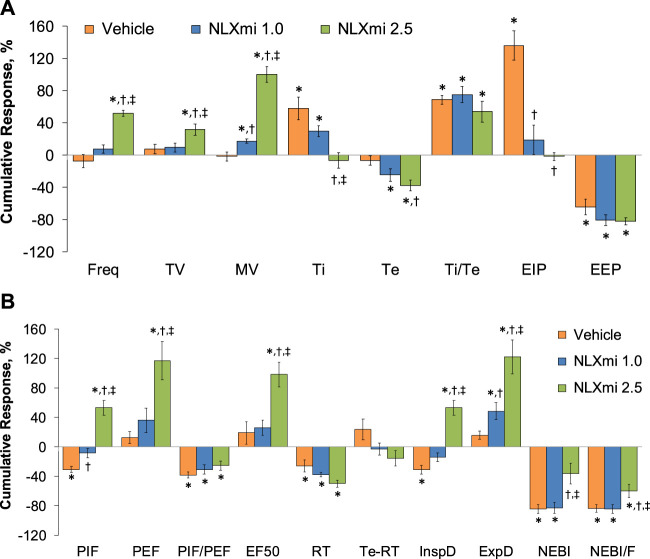
Cumulative responses (expressed as % change from Pre-values) [panels **(A,B)**] recorded over the 10-min period following the injection vehicle or NLXmi (1.0 or 2.5 mg/kg, IV) in male rats that received a prior injection of fentanyl (75 μg/kg, IV). There were four rats in each group. The data are presented as the mean ± SEM. *p < 0.05, significant change from Pre-values. ^†^ *p* < 0.05, NLXmi 1.0 or 2.5 versus vehicle. ^‡^ *p* < 0.05, NLXmi 2.5 versus NLXmi 1.0. **ANOVA statistics:** Freq: F_(2,9)_ = 63.9 and *p* = 0.000; TV: F_(2,9)_ = 4.6 and *p* = 0.042; MV: F_(2,9)_ = 65.0 and *p* = 0.000; Ti: F_(2,9)_ = 9.3 and *p* = 0.007; Te: F_(2,9)_ = 15.6 and *p* = 0.001; Ti/Te: F_(2,9)_ = 1.9 and *p* = 0.203; EIP: F_(2,9)_ = 23.8 and *p* = 0.000; EEP: F_(2,9)_ = 1.9 and *p* = 0.207; PIF: F_(2,9)_ = 27.4 and *p* = 0.000; PEF: F_(2,9)_ = 11.0 and *p* = 0.004; PIF/PEF: F_(2,9)_ = 1.0 and *p* = 0.398; EF_50_: F_(2,9)_ = 9.2 and *p* = 0.007; RT: F_(2,9)_ = 6.3 and *p* = 0.020; Te-RT: F_(2,9)_ = 1.2 and *p* = 0.358; InspD: F_(2,9)_ = 34.4 and *p* = 0.000; ExpD: F_(2,9)_ = 11.2 and *p* = 0.004; NEBI: F_(2,9)_ = 7.8 and *p* = 0.011; NEBI/F: F_(2,9)_ = 4.1 and *p* = 0.550.

The cumulative responses (expressed as % change from pre-values) recorded over the 10-min period after the injection of vehicle or NLXmi (1.0 or 2.5 mg/kg, IV) in rats that received a prior injection of fentanyl (75 μg/kg, IV) are summarized in Figure 11. As shown in **Panel A**, the 1.0 mg/kg dose of NLXmi elicited a minor increase in MV (i.e., a greater increase than in vehicle-injected rats), whereas it substantially diminished the increase in EIP observed in the vehicle-treated rats. As shown in **Panel B**, the 1.0 mg/kg dose of NLXmi diminished the decrease in PIF seen in vehicle-treated rats and produced a relatively robust increase in expiratory drive (ExpD, TV/Te). In contrast, the 2.5 mg/kg dose of NLXmi elicited pronounced effects on Freq, TV, MV, Ti, Te, and EIP but not on Ti/Te or EEP (**Panel A**) and on PIF, PEF, PIF/PEF, EF_50_, relaxation time, inspiratory drive (InspD, TV/Ti), expiratory drive (ExpD, TV/Te), NEBI, and NEBI/Freq but not on relaxation time or Te-RT (**Panel B**).

### Summary of the ventilatory responses in female rats

**FIGURE 12:**
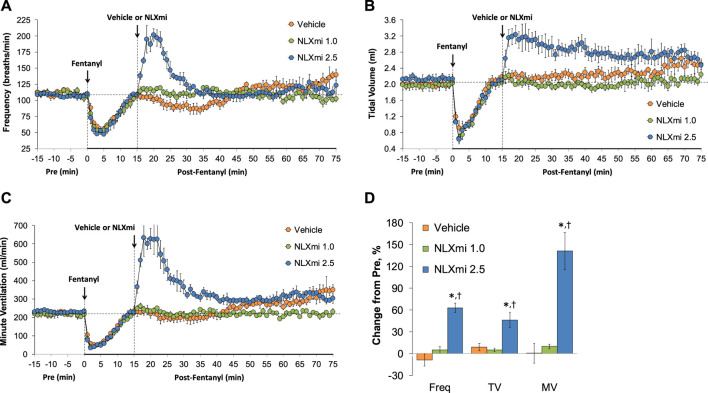
Frequency of breathing **(Panel A)**, tidal volume **(Panel B)**, and minute ventilation **(Panel C)** before (Pre) and after the injection of fentanyl (75 μg/kg, IV) and subsequent injection of vehicle or NLXmi (1.0 or 2.5 mg/kg, IV) in female rats. The data in **Panel D** show the cumulative responses (expressed as % change from Pre-values) recorded over the 10-min period following the injection of vehicle or NLXmi (1.0 or 2.5 mg/kg, IV) in female rats that received a prior injection of fentanyl (75 μg/kg, IV). There were four rats in each group. The data are presented as the mean ± SEM. *p < 0.05, significant change from Pre-values. ^†^ *p* < 0.05, NLXmi 2.5 versus NLXmi 1.0 or vehicle.

As summarized in **Panel A** of Supplemental Figure S4, there were no between-group differences in the declines observed in Freq, TV, and MV elicited by the injection of fentanyl (75 μg/kg, IV) in the female rats that would subsequently receive vehicle or NLXmi (1.0 or 2.5 mg/kg, IV) (*p* > 0.05 for all comparisons). As summarized in **Panel B**, the effects of fentanyl had fully resolved by 15 min after injection (*p* > 0.05 for all comparisons). As shown in Figure 12, the injection of fentanyl (75 μg/kg, IV) elicited pronounced decreases in Freq (**Panel A**), TV (**Panel B**), and MV (**Panel C**) in the three groups of female rats that had fully resolved within 10–12 min. The subsequent administration of vehicle or NLXmi (1.0 mg/kg, IV) at the 15-min post-fentanyl time-point did not elicit immediate changes in Freq, TV, or MV. In contrast, the injection of the 2.5 mg/kg dose of NLXmi elicited a prompt increase in Freq of approximately 10 min in duration and a prompt and sustained increase in TV. The changes in Freq and TV resulted in a prompt increase in MV of approximately 25–30 min in duration. **Panel D** summarizes the total cumulative responses recorded over the 10-min period following the injection vehicle or NLXmi (1.0 or 2.5 mg/kg, IV) in female rats that received a prior injection of fentanyl (75 μg/kg, IV). As can be seen, only the 2.5 mg/kg dose of NLXmi elicited significant responses.

## Discussion

### NLXmi-induced reversal of fentanyl-induced sedation

The lack of effect of the 1.0 and 2.5 mg/kg doses of NLXmi on resting ventilatory parameters and the behaviors of vehicle-treated rats (no visible signs of arousal, for example) are consistent with previous findings and suggest that central opioidergic systems potentially accessed by NLXmi are not tonically active (Lewanowitsch and Irvine, 2002; 2003; Lewanowitsch et al., 2006; Leppert, 2010; Yamamoto and Sugimoto, 2010; Mori et al., 2013; Henderson et al., 2014; Belltall et al., 2022). The finding that the 1.0 mg/kg dose of NLXmi did not cause arousal in the fentanyl-injected rats is most likely because insufficient amounts of the opioid receptor antagonist reached key areas in the brain. However, the distinct arousal elicited by the 2.5 mg/kg dose of NLXmi in the fentanyl-injected rats (reversal of sedation evidenced by restoration of the righting reflex) certainly suggests that this systemic dose provided sufficient amounts of NLXmi to be centrally active. The question arises as to whether this arousal is directly linked to the observed ventilatory responses that will be addressed below. The two processes may not be linked since the injection of the 2.5 mg/kg dose of NLXmi caused pronounced ventilatory responses in fentanyl-treated rats, whereas these ventilatory responses were not associated with increases in either NEBI or NEBI/Freq, which are hallmarks of ventilatory instability (Getsy et al., 2014; Gaston et al., 2021; Getsy et al., 2022c; Getsy et al., 2022d; Getsy et al., 2022e; Lewis et al., 2022). More specifically, it appeared that the 2.5 mg/kg dose of NLXmi merely restored the behavioral status of the fentanyl-treated rats but caused unexpectedly dramatic changes in ventilation, which suggests the existence of an inhibitory opioid receptor system in ventilatory control pathways. As stated, the 2.5 mg/kg dose of NLXmi elicited minor responses in naïve rats. As such, the observed increase in Freq, TV, and MV elicited by the 2.5 mg/kg of NLXmi in the fentanyl-treated rats is likely due to the direct antagonism of opioid receptors rather than because of unexplained side effects of NLXmi.

### Ventilatory responses elicited by fentanyl

The usual analysis of the effects of drugs on breathing relies upon the recording of Freq, TV, and MV (multiplicative index of Freq and TV). The reporting of Ti and Te and respiratory quotient (Ti/Te) is much less frequent, despite their obvious importance to understanding how inspiratory and expiratory networks are affected by drugs. Moreover, the reporting of EIP and EEP is rare, even though these parameters provide key information as to how drugs such as opioids affect predominantly brainstem mechanisms controlling pauses between inspiration and expiration (Getsy et al., 2014; Gaston et al., 2021; Getsy et al., 2022c; d,e; Lewis et al., 2022). Many other key parameters, such as inspiratory and expiratory drives, PIF, PEF, EF_50_, relaxation time, expiratory delay (Te-RT), and NEBI, are similarly underused parameters of value to the understanding of respiratory physiology and the effects of drugs and disease processes on ventilatory processes. Our laboratory has described the effects of a variety of challenges, including opioid administration, on the above parameters to provide a more comprehensive understanding of the mechanisms of action of opioids and the processes by which a series of novel drugs affect opioid-induced respiratory depression (OIRD) (Henderson et al., 2013; 2014; Gaston et al., 2020; Getsy et al., 2014; Gaston et al., 2021; Jenkins et al., 2021; Getsy et al., 2022a; Getsy et al., 2022b; Getsy et al., 2022c; Getsy et al., 2022d; Getsy et al., 2022e; Lewis et al., 2022; Seckler et al., 2022).

The present study shows that the injection of fentanyl (75 μg/kg, IV) caused pronounced decreases in Freq, TV, and MV that had fully resolved within 15 min. However, it was evident that fentanyl was still having important effects on ventilatory processes at this and later time-points. To begin with, the findings that Ti and EIP were increased whereas Te and EEP were decreased at 15 min demonstrate that monitoring Freq alone is an inadequate index of the effects of fentanyl. The mechanisms by which fentanyl differentially affects inspiratory and expiratory parameters are multi-factorial (Getsy et al., 2014; 2022a; b; Henderson et al., 2014; Jenkins et al., 2021) and need further investigation, especially in studies designed to develop drugs that may combat the unwanted effects of fentanyl (i.e., increases in Ti and EIP) as opposed to potentially beneficial effects (e.g., shortening of Te and EEP). Although the effects of fentanyl on TV had resolved within 15 min, other volume-related parameters were still altered at this time. For example, PIF was markedly depressed, whereas PEF and EF_50_ were slightly augmented, which again highlights the differential effects of fentanyl on inspiration and expiration. Further highlighting the excitatory effects of fentanyl on expiratory processes, it was evident that the opioid markedly diminished relaxation time (decay of expiration to 36% maximum), an effect that was fully evident at the 15 min post-injection time. This suggests that fentanyl actively enhances what is usually a passive expulsion of air (Getsy et al., 2014; Henderson et al., 2014). As a result of the differential magnitude of the reductions in Te and relaxation time, expiratory delay (Te-RT, see Supplemental Figure S1) was actually increased initially by fentanyl, whereas the respiratory delay was back to pre-fentanyl levels at 15 min. As such, the time taken following the completion of relaxation time (active expulsion of air) to when inspiration takes place is lengthened for several minutes by fentanyl. As a result of the changes in TV, Ti, and Te, it was evident that fentanyl elicited pronounced decreases in inspiratory drive (TV/Ti) and expiratory drive (TV/Te). However, inspiratory drive was still depressed at 15 min post-fentanyl, whereas expiratory drive was elevated at this time (both changes due to increases in Ti and decreases in Te since TV was back to pre-injection values). Finally, as expected (Getsy et al., 2014; 2022a; b; Henderson et al., 2014; Jenkins et al., 2021), the injection of fentanyl caused a pronounced increase in NEBI and NEBI/Freq that persisted for approximately 5 min. The ability of fentanyl to destabilize breathing is a vital effect of this opioid and consists of increases in both the number and duration of apneas and disordered breathing events (Getsy et al., 2014). Our previous evidence that the effects of 25–75 μg/kg doses of fentanyl on arterial blood–gas chemistry (pH, pCO_2_, and pO_2_ sO_2_) and alveolar gas exchange (alveolar–arterial gradient) had fully resolved upon the recovery of tidal volume in freely-moving male rats (Henderson et al., 2014; Jenkins et al., 2021) suggests that the two putative counter-balancing systems activated by fentanyl have together caused a “normalization” of arterial blood–gas chemistry.

### Ventilatory effects of NLXmi

The administration of NLXmi elicits minor observable changes in behavior and cardiorespiratory parameters in naïve rats and mice, whereas it exerts important effects on the analgesic, behavioral, and cardiorespiratory effects of opioids, including fentanyl (Lewanowitsch and Irvine, 2002; 2003; Lewanowitsch et al., 2006; Leppert, 2010; Yamamoto and Sugimoto, 2010; Mori et al., 2013; Henderson et al., 2014; Belltall et al., 2022). Although the available evidence suggests that NLXmi is peripherally restricted, this evidence stems from behavioral/pharmacological studies, and it should be noted that there is no direct evidence as to the blood–brain penetrability of NLXmi and, therefore, the amounts of the drug that enter the brain (Leppert, 2010; Henderson et al., 2014). As mentioned, the injection of the 1.0 mg/kg dose of NLXmi did not arouse the fentanyl-treated rats when given at 15 min post-fentanyl administration but had some important effects on some ventilatory parameters. More specifically, this lower dose of NLXmi elicited no or minor effects on Freq, TV, MV, Te, Ti/Te, EEP, EF_50_, relaxation time, expiratory delay (Te-RT), NEBI, and NEBI/Freq. In contrast, the 1.0 mg/kg dose of NLXmi caused immediate decreases in Ti and EIP (that were both elevated by fentanyl) and immediate increases in PIF and inspiratory drive (that were both decreased by fentanyl) while also increasing PEF (values at pre-fentanyl injection levels). Since this dose of NLXmi did not arouse the rats, it is possible that these effects of NLXmi are due to actions in the periphery (Henderson et al., 2014), although this cannot be affirmed. A major set of findings was that the 2.5 mg/kg dose of NLXmi elicits prompt and sustained arousal of the sedative effects of fentanyl and some pronounced effects on ventilatory parameters. It would, therefore, seem possible that, despite evidence that NLXmi is peripherally restricted (Lewanowitsch and Irvine, 2002; Lewanowitsch and Irvine, 2003; Lewanowitsch et al., 2006; Leppert, 2010; Yamamoto and Sugimoto, 2010; Mori et al., 2013; Henderson et al., 2014; Belltall et al., 2022), the injection of the 2.5 mg/kg dose allows enough NLXmi to cross the blood–brain barrier to be functionally effective in relevant brain structures. Alternatively, the higher dose of NLXmi may be able to exert its effects via sites such as the area postrema and anteroventral region of the third ventricle that are devoid of a blood–brain barrier (Johnson and Gross, 1993; Lewis et al., 1999). Our findings may prompt studies to provide direct evidence regarding the possible penetration of systemically injected NLXmi into deep brain structures. The administration of 2.5 mg/kg NLXmi elicited substantial increases in Freq, TV, and MV when administered at 15 min post-fentanyl, a time in which resting values had returned to pre-fentanyl levels. The ability of NLXmi to elicit a substantial and long-lasting increase in TV speaks in particular to our concept that fentanyl had recruited two active counter-balancing pathways controlling breathing, one being depressant and involving opioid receptors, and the other (as yet unidentified) being excitatory and uncovered after the blockade of opioid receptors. The ability of the 2.5 mg/kg dose of NLXmi to produce robust increases in Freq would not be expected on the basis that resting Freq had recovered from fentanyl but would be expected when considering that fentanyl was still affecting both Ti and Te. Taking the changes in TV, Ti, and Te into account, it was evident that the higher dose of NLXmi elicited substantial increases in inspiratory drive (TV/Ti) from a decreased fentanyl-induced baseline and expiratory drive (TV/Te) from a fentanyl-induced increase in baseline. As such, it is clear that the blockade of an on-going opioid receptor-dependent ventilatory depressant pathway (most likely in the brain) unveils the existence of a remarkably effective excitatory pathway that is somehow triggered by fentanyl via the direct pharmacological action of the opioids or indirectly from the induced hypoxemia, for example.

The disparate effects of the higher dose of NLXmi on EIP and EEP are informative in that NLXmi elicited a dramatic decrease in the elevated levels of EIP caused by fentanyl, whereas it minimally affected EEP, which was depressed by fentanyl. It would seem evident that opioid receptors play dramatically different roles in the control of EIP and EEP and, therefore, in the switching of inspiratory to expiratory events and *vice versa*. Consistent with the observed effects of NLXmi on TV, the injection of the higher dose of NLXmi elicited substantial increases in PIF (from a fentanyl-induced depression at baseline) and in PEF and EF_50_ (from fentanyl-induced increases at baseline). It would appear that the activities of the counter-balancing systems result in a substantial depression of PIF and a lesser enhancement of PEF and EF_50_. Regardless, blocking the opioid receptor inhibitory system results in the expression of the excitatory system that drives robust increases in PIF, PEF, and EF_50_. The finding that the higher dose of NLXmi did not affect fentanyl-induced decreases in relaxation time is problematic for the idea that fentanyl acts only via activation of opioid (predominantly) μ-receptors (Comer and Cahill, 2019; Moss et al., 2020; Vo et al., 2021; Kelly et al., 2023). Indeed, fentanyl interacts directly with an array of K^+^-channels (Lee et al., 1995; Montandon et al., 2016; Tschirhart et al., 2019; Tschirhart and Zhang, 2020), has high affinity for α_1A_ and α_1B_ adrenoceptors and dopamine D4.4 and D1 receptors, and blocks vesicular monoamine transporter 2 (Torralva et al., 2020). Moreover, remifentanil directly activates human glutamatergic N-methyl-D-aspartate (NMDA) receptors expressed in *Xenopus laevis* oocytes (Hahnenkamp et al., 2004). Whether these functional proteins are integral to the activity of the excitatory pathway unveiled by NLXmi remains to be established. It follows that the relatively equivalent changes in Te and relaxation time elicited by the 2.5 mg/kg dose of NLXmi resulted in minimal changes in expiratory delay (Te-RT). Finally, the lack of effect of the higher dose of NLXmi on the suppression of NEBI and NEBI/Freq induced by fentanyl (i.e., the synthetic opioid unexpectedly diminished the occurrence of non-eupneic breathing events) again points to the possibility that these effects of fentanyl do not involve opioid receptors susceptible to blockade by NLXmi. The relative roles of μ-, δ-, and κ-opioid receptors in the pharmacological actions of NLXmi have been addressed, and the available evidence shows that it has a much higher affinity/efficacy for μ-opioid receptors than δ- and κ-opioid receptors, with the order of potency being μ- > δ- > κ-opioid receptors (Lewanowitsch and Irvine, 2003; Leppert, 2010). As such, we tentatively suggest that the ability of the higher dose of NLXmi to elicit its effects primarily involves the blockade of central μ-opioid receptors.

### Study limitations

An important conclusion from this study is that fentanyl appeared to have induced two on-going counter-balancing systems that control breathing: one inhibitory and one excitatory. The ability of fentanyl to elicit excitatory effects on breathing has been recently demonstrated in unanesthetized female goats. The stimulatory effects of fentanyl on breathing were associated with an increased rate of rise of the diaphragm muscle activity and increased activation of upper airway, intercostal, and abdominal muscles (Neumueller et al., 2023). On the basis of the effects of the higher dose of NLXmi, it appears that one is an opioid receptor-dependent inhibitory system. Future studies need to provide more information as to where in the brain this inhibitory system is located and whether μ- and or δ-opioid receptors, which are the primary targets for NLXmi (Henderson et al., 2014), are integral to the system. An important limitation of this study is that we have not provided evidence regarding the nature of the excitatory system. On the basis of their potential involvement, we are determining whether NMDA receptors (Hoffmann et al., 2003) or S-nitrosothiols (Getsy et al., 2022b; Getsy et al., 2022f) are integral components of the excitatory system. Key evidence supporting these future studies is that the co-administration of NMDA receptor antagonists (ketamine and dextromethorphan) increases opioid-induced respiratory depression (Hoffmann et al., 2003) and that the endogenous S-nitrosothiol, S-nitroso-L-cysteine, stereoselectively blunts the adverse effects of fentanyl (Getsy et al., 2022b) and morphine (Getsy et al., 2022a) on breathing and arterial blood–gas chemistry. A limitation of our study pertains to the doses of NLXmi used. It would seem imperative to determine whether higher doses (e.g., 5 or 10 mg/kg) elicit even greater responses than the 1.0 or 2.5 mg/kg doses used here. Moreover, since opioids exert qualitatively and quantitatively different effects on ventilatory systems in female subjects than in male subjects (Dahan et al., 1998; Hosseini et al., 2011), it is important to determine whether there is a sex-dependent difference in the ability of fentanyl to recruit the inhibitory and excitatory systems. Another limitation is that these studies were performed using a single opioid at a single dose. The drug and dose were chosen to produce a profound respiratory depression with a duration short enough to avoid mortality and allow full spontaneous recovery of minute ventilation. If the non-opioid receptor response is induced by fentanyl binding to another receptor with different ligand binding determinants, then an opioid with a dissimilar chemical structure, like morphine, might produce a different response; however, if the response is triggered by a physiological response to fentanyl binding, such as hypoxemia, or a biochemical response, such as the activation of β-arrestin, then the response is more likely to be similar.

## Conclusion

Since fentanyl is a widely used in-hospital opioid and is playing a major role in the current opioid crisis (van der Schier et al., 2014; Algera et al., 2019; Imam et al., 2020; Arendt, 2021), it is vital to quickly determine the full efficacy profile of NLXmi and other opioid antagonists with respect to overcoming opioid-induced respiratory depression. In therapeutic and recreational use, fentanyl is often co-administered with other drugs, and if these drugs interact with the induced excitatory system, then the combined respiratory depression may be more than additive and harder to reverse. The first conclusion from these studies is that simply analyzing the effects of fentanyl on Freq, TV, and MV is inadequate for addressing the full profile of fentanyl actions on breathing. Clear examples are that whereas Freq had returned to pre-fentanyl levels at 15 min, Ti and EIP were increased and Te and EEP were reduced. Measuring Freq alone would lead to the false conclusion that the effects of fentanyl on respiratory timing had subsided, whereas monitoring the other parameters would give a clearer picture of the mechanism of action of fentanyl on respiratory networks in the central and peripheral nervous systems. The second conclusion from these studies is that the injection of the higher dose of NLXmi did not elicit behavioral or ventilatory responses in vehicle-injected rats, whereas the injection of NLXmi elicited dramatic increases in TV in fentanyl-injected rats at a time that resting TV had returned to pre-fentanyl levels. This suggests that fentanyl has induced on-going counter-balancing systems controlling TV and that the blockade of the μ-opioid receptor-dependent inhibitory system unveils the excitatory system. The ability of the higher dose of NLXmi to reverse on-going effects on an array of parameters, such as PIF, also strongly implicates the role of opioid receptor-dependent pathways in the respiratory depressant effects of fentanyl. The inability of NLXmi to reverse many of the effects of fentanyl raises the possibility that this synthetic opioid recruits non-opioid receptor-dependent processes that stimulate breathing. It must be stated that the ability of the higher dose of NLXmi to elicit its ventilatory responses may involve actions on opioidergic systems within brain regions (i.e., sites within the brainstem) that are not necessarily accessed/affected by fentanyl. Again, these actions of the higher doses of NLXmi are likely to be within the brain, as the lower dose elicited only minimal responses. The ability of fentanyl to both excite and inhibit ventilatory parameters suggests that the activation of μ-opioid receptors triggers central pathways that subserve the activation and suppression of breathing, respectively (Neumueller et al., 2023). Our on-going studies are finding that a 1.0 mg/kg dose of naloxone, naloxonazine, or naltrexone elicits very similar behavioral (e.g., reversal of sedation) and ventilatory responses in fentanyl-treated male and female rats to those produced by the 2.5 mg/kg dose of NLXmi. The data from these centrally penetrant opioid receptor antagonists support the contention that the actions of the 2.5 mg/kg dose of NLXmi may be due to central penetration. Moreover, they suggest that the 1.0 mg/kg dose of NLXmi caused changes to the actions of fentanyl by acting at peripheral sites of action since this dose did not arouse the rats.

## Data Availability

The raw data supporting the conclusion of this article will be made available by the authors, without undue reservation.

## References

[B1] Algera M. H. Kamp J. van der Schrier R. van Velzen M. Niesters M. Aarts L. (2019). Opioid-induced respiratory depression in humans: a review of pharmacokinetic-pharmacodynamic modelling of reversal. Br. J. Anaesth. 122, e168–e179. 10.1016/j.bja.2018.12.023 30915997

[B2] Al-Hasani R. Bruchas M. R. (2011). Molecular mechanisms of opioid receptor-dependent signaling and behavior. Anesthesiology 115, 1363–1381. 10.1097/ALN.0b013e318238bba6 22020140 PMC3698859

[B3] Arendt F. (2021). The opioid-overdose crisis and fentanyl: the role of online information seeking via internet search engines. Health Commun. 36, 1148–1154. 10.1080/10410236.2020.1748820 32285691

[B4] Armenian P. Vo K. T. Barr-Walker J. Lynch K. L. (2018). Fentanyl, fentanyl analogs and novel synthetic opioids: a comprehensive review. Neuropharmacology 134, 121–132. 10.1016/j.neuropharm.2017.10.016 29042317

[B5] Baby S. M. Gruber R. Discala J. Puskovic V. Jose N. Cheng F. (2021). Systemic administration of tempol attenuates the cardiorespiratory depressant effects of fentanyl. Front. Pharmacol. 12, 690407. 10.3389/fphar.2021.690407 34248639 PMC8260831

[B6] Baby S. M. Gruber R. B. Young A. P. MacFarlane P. M. Teppema L. J. Lewis S. J. (2018). Bilateral carotid sinus nerve transection exacerbates morphine-induced respiratory depression. Eur. J. Pharmacol. 834, 17–29. 10.1016/j.ejphar.2018.07.018 30012498 PMC6091892

[B7] Baby S. M. May W. J. Young A. P. Wilson D. G. Getsy P. M. Coffee G. A. (2023). L-cysteine ethylester reverses the adverse effects of morphine on breathing and arterial blood-gas chemistry while minimally affecting antinociception in unanesthetized rats. Biomed. Pharmacother. 171, 116081. revision. 10.1016/j.biopha.2023.116081 PMC10922989 38219385

[B8] Baker L. Ratka A. (2002). Sex-specific differences in levels of morphine, morphine-3-glucuronide, and morphine antinociception in rats. Pain 95, 65–74. 10.1016/s0304-3959(01)00376-1 11790468

[B9] Belltall A. Mazzinari G. Diaz-Cambronero O. Eroles P. Argente Navarro M. P. (2022). Antagonists of the mu-opioid receptor in the cancer patient: fact or fiction? Curr. Oncol. Rep. 24, 1337–1349. 10.1007/s11912-022-01295-z 35648340 PMC9474368

[B10] Birdsong W. T. Williams J. T. (2020). Recent progress in opioid research from an electrophysiological perspective. Mol. Pharmacol. 98, 401–409. 10.1124/mol.119.119040 32198208 PMC7562972

[B11] Blomqvist K. J. Viisanen H. Ahlström F. H. G. Jokinen V. Sidorova Y. A. Suleymanova I. (2020). Morphine-3-glucuronide causes antinociceptive cross-tolerance to morphine and increases spinal substance P expression. Eur. J. Pharmacol. 875, 173021. 10.1016/j.ejphar.2020.173021 32112778

[B12] Christrup L. L. (1997). Morphine metabolites. Acta Anaesthesiol. Scand. 41, 116–122. 10.1111/j.1399-6576.1997.tb04625.x 9061094

[B13] Comer S. D. Cahill C. M. (2019). Fentanyl: receptor pharmacology, abuse potential, and implications for treatment. Neurosci. Biobehav Rev. 106, 49–57. 10.1016/j.neubiorev.2018.12.005 30528374 PMC7233332

[B14] Dahan A. Sarton E. Teppema L. Olievier C. (1998). Sex-related differences in the influence of morphine on ventilatory control in humans. Anesthesiology 88, 903–913. 10.1097/00000542-199804000-00009 9579498

[B15] Ekblom M. Gårdmark M. Hammarlund-Udenaes M. (1993). Pharmacokinetics and pharmacodynamics of morphine-3-glucuronide in rats and its influence on the antinociceptive effect of morphine. Biopharm. Drug Dispos. 14, 1–11. 10.1002/bdd.2510140102 8427941

[B16] Epstein M. A. Epstein R. A. (1978). A theoretical analysis of the barometric method for measurement of tidal volume. Respir. Physiol. 32, 105–120. 10.1016/0034-5687(78)90103-2 625610

[B17] Epstein R. A. Epstein M. A. Haddad G. G. Mellins R. B. (1980). Practical implementation of the barometric method for measurement of tidal volume. J. Appl. Physiol. 49, 1107–1115. 10.1152/jappl.1980.49.6.1107 7440298

[B18] Gaston B. Baby S. M. May W. J. Young A. P. Grossfield A. Bates J. N. (2021). D-Cystine di(m)ethyl ester reverses the deleterious effects of morphine on ventilation and arterial blood gas chemistry while promoting antinociception. Sci. Rep. 11, 10038. 10.1038/s41598-021-89455-2 33976311 PMC8113454

[B19] Getsy P. M. Baby S. M. Gruber R. B. Gaston B. Lewis T. H. J. Grossfield A. (2022b). S-Nitroso-L-Cysteine stereoselectively blunts the deleterious effects of fentanyl on breathing while augmenting antinociception in freely-moving rats. Front. Pharmacol. 13, 892307. 10.3389/fphar.2022.892307 35721204 PMC9199495

[B20] Getsy P. M. Baby S. M. May W. J. Bates J. N. Ellis C. R. Feasel M. G. (2022c). L-cysteine methyl ester overcomes the deleterious effects of morphine on ventilatory parameters and arterial blood-gas chemistry in unanesthetized rats. Front. Pharmacol. 13, 968378. 10.3389/fphar.2022.968378 36249760 PMC9554613

[B21] Getsy P. M. Baby S. M. May W. J. Lewis T. H. J. Bates J. N. Hsieh Y. H. (2022a). L-NAC reverses of the adverse effects of fentanyl infusion on ventilation and blood-gas chemistry. Biomed. Pharmacother. 153, 113277. 10.1016/j.biopha.2022.113277 35724513 PMC9458628

[B22] Getsy P. M. Baby S. M. May W. J. Young A. P. Gaston B. Hodges M. R. (2022d). D-cysteine ethyl ester reverses the deleterious effects of morphine on breathing and arterial blood-gas chemistry in freely-moving rats. Front. Pharmacol. 13, 883329. 10.3389/fphar.2022.883329 35814208 PMC9260251

[B23] Getsy P. M. Coffee G. A. Lewis S. J. (2023b). Loss of ganglioglomerular nerve input to the carotid body impacts the hypoxic ventilatory response in freely-moving rats. Front. Physiol. 14, 1007043. 10.3389/fphys.2023.1007043 37008015 PMC10060956

[B24] Getsy P. M. Davis J. Coffee G. A. Lewis T. H. J. Lewis S. J. (2023a). Hypercapnic signaling influences hypoxic signaling in the control of breathing in C57BL6 mice. J. Appl. Physiol. 134, 1188–1206. 10.1152/japplphysiol.00548.2022 36892890 PMC10151047

[B25] Getsy P. M. Davis J. Coffee G. A. May W. J. Palmer L. A. Strohl K. P. (2014). Enhanced non-eupneic breathing following hypoxic, hypercapnic or hypoxic-hypercapnic gas challenges in conscious mice. Respir. Physiol. Neurobiol. 204, 147–159. 10.1016/j.resp.2014.09.006 25242462 PMC4252883

[B26] Getsy P. M. Young A. P. Bates J. N. Baby S. M. Seckler J. M. Grossfield A. (2022f). S-nitroso-L-cysteine stereoselectively blunts the adverse effects of morphine on breathing and arterial blood gas chemistry while promoting analgesia. Biomed. Pharmacother. 153, 113436. 10.1016/j.biopha.2022.113436 36076552 PMC9464305

[B27] Getsy P. M. Young A. P. Grossfield A. Seckler J. M. Wilson C. G. Gaston B. (2022e). D-cysteine ethyl ester and D-cystine dimethyl ester reverse the deleterious effects of morphine on arterial blood-gas chemistry and Alveolar-arterial gradient in anesthetized rats. Resp. Physiol. Neurobiol. 302, 103912. 10.1016/j.resp.2022.103912 PMC9588175 35447347

[B28] Hahnenkamp K. Nollet J. Van Aken H. K. Buerkle H. Halene T. Schauerte S. (2004). Remifentanil directly activates human N-methyl-D-aspartate receptors expressed in *Xenopus laevis* oocytes. Anesthesiology 100, 1531–1537. 10.1097/00000542-200406000-00028 15166575

[B29] Hanna M. H. Peat S. J. Knibb A. A. Fung C. (1991). Disposition of morphine-6-glucuronide and morphine in healthy volunteers. Br. J. Anaesth. 66, 103–107. 10.1093/bja/66.1.103 1997044

[B30] Henderson F. May W. J. Gruber R. B. Discala J. F. Puskovic V. Young A. P. (2014). Role of central and peripheral opiate receptors in the effects of fentanyl on analgesia, ventilation and arterial blood-gas chemistry in conscious rats. Respir. Physiol. Neurobiol. 191, 95–105. 10.1016/j.resp.2013.11.005 24284037 PMC4391496

[B31] Henderson F. May W. J. Gruber R. B. Young A. P. Palmer L. A. Gaston B. (2013). Low-dose morphine elicits ventilatory excitant and depressant responses in conscious rats: role of peripheral μ-opioid receptors. Open J. Mol. Integr. Physiol. 3, 111–124. 10.4236/ojmip.2013.33017 24900948 PMC4041292

[B32] Hoffmann V. L. Vermeyen K. M. Adriaensen H. F. Meert T. F. (2003). Effects of NMDA receptor antagonists on opioid-induced respiratory depression and acute antinociception in rats. Pharmacol. Biochem. Behav. 74, 933–941. 10.1016/s0091-3057(03)00020-0 12667908

[B33] Hosseini M. Taiarani Z. Hadjzadeh M. A. Salehabadi S. Tehranipour M. Alaei H. A. (2011). Different responses of nitric oxide synthase inhibition on morphine-induced antinociception in male and female rats. Pathophysiology 18, 143–149. 10.1016/j.pathophys.2010.05.004 20558049

[B34] Imam M. Z. Kuo A. Smith M. T. (2020). Countering opioid-induced respiratory depression by non-opioids that are respiratory stimulants. F1000Res 9, F1000 Faculty Rev-91. Faculty Rev-91. 10.12688/f1000research.21738.1 PMC7008602 32089833

[B35] Jenkins M. W. Khalid F. Baby S. M. May W. J. Young A. P. Bates J. N. (2021). Glutathione ethyl ester reverses the deleterious effects of fentanyl on ventilation and arterial blood-gas chemistry while prolonging fentanyl-induced analgesia. Sci. Rep. 11, 6985. 10.1038/s41598-021-86458-x 33772077 PMC7997982

[B36] Johnson A. K. Gross P. M. (1993). Sensory circumventricular organs and brain homeostatic pathways. FASEB J. 7, 678–686. 10.1096/fasebj.7.8.8500693 8500693

[B37] Kelly E. Sutcliffe K. Cavallo D. Ramos-Gonzalez N. Alhosan N. Henderson G. (2023). The anomalous pharmacology of fentanyl. Br. J. Pharmacol. 180, 797–812. 10.1111/bph.15573 34030211

[B38] Lalley P. M. (2003). Mu-opioid receptor agonist effects on medullary respiratory neurons in the cat: evidence for involvement in certain types of ventilatory disturbances. Am. J. Physiol. Regul. Integr. Comp. Physiol. 285, R1287–R1304. 10.1152/ajpregu.00199.2003 12881202

[B39] Lee T. Y. Fu M. J. Lui P. W. Chan S. H. (1995). Involvement of potassium and calcium channels at the locus coeruleus in fentanyl-induced muscular rigidity in the rat. Neurosci. Lett. 199, 195–198. 10.1016/0304-3940(95)12049-a 8577396

[B40] Leppert W. (2010). The role of opioid receptor antagonists in the treatment of opioid-induced constipation: a review. Adv. Ther. 27, 714–730. 10.1007/s12325-010-0063-0 20799006

[B41] Lewanowitsch T. Irvine R. J. (2002). Naloxone methiodide reverses opioid-induced respiratory depression and analgesia without withdrawal. Eur. J. Pharmacol. 445, 61–67. 10.1016/s0014-2999(02)01715-6 12065195

[B42] Lewanowitsch T. Irvine R. J. (2003). Naloxone and its quaternary derivative, naloxone methiodide, have differing affinities for mu, delta, and kappa opioid receptors in mouse brain homogenates. Brain Res. 964, 302–305. 10.1016/s0006-8993(02)04117-3 12576191

[B43] Lewanowitsch T. Miller J. H. Irvine R. J. (2006). Reversal of morphine, methadone and heroin induced effects in mice by naloxone methiodide. Life Sci. 78, 682–688. 10.1016/j.lfs.2005.05.062 16102783

[B44] Lewis S. J. Whalen E. J. Beltz T. G. Johnson A. K. (1999). Role of the anterior region of the third ventricle in the cardiovascular responses produced by systemic injection of a nitric oxide synthase inhibitor. Brain Res. 830, 191–194. 10.1016/s0006-8993(99)01351-7 10350574

[B45] Lewis T. H. J. May W. J. Young A. P. Bates J. N. Baby S. M. Getsy P. M. (2022). The ventilatory depressant actions but not the antinociceptive effects of morphine are blunted in rats receiving intravenous infusion of L-cysteine ethyl ester. Biomed. Pharmacother. 156, 113939. 10.1016/j.biopha.2022.113939 36411626 PMC9682160

[B46] Lomask M. (2006). Further exploration of the Penh parameter. Exp. Toxicol. Pathol. 57 (Suppl. 2), 13–20. 10.1016/j.etp.2006.02.014 16638630

[B47] Marchette R. C. N. Carlson E. R. Frye E. V. Hastings L. E. Vendruscolo J. C. M. Mejias-Torres G. (2023). Heroin- and fentanyl-induced respiratory depression in a rat plethysmography model: potency, tolerance, and sex differences. J. Pharmacol. Exp. Ther. 385, 117–134. 10.1124/jpet.122.001476 36828628 PMC10108442

[B48] May C. N. Dashwood M. R. Whitehead C. J. Mathias C. J. (1989). Differential cardiovascular and respiratory responses to central administration of selective opioid agonists in conscious rabbits: correlation with receptor distribution. Br. J. Pharmacol. 98, 903–913. 10.1111/j.1476-5381.1989.tb14620.x 2556206 PMC1854750

[B49] May W. J. Gruber R. B. Discala J. F. Puskovic V. Henderson F. Palmer L. A. (2013a). Morphine has latent deleterious effects on the ventilatory responses to a hypoxic challenge. Open J. Mol. Integr. Physiol. 3, 166–180. 10.4236/ojmip.2013.34022 25045593 PMC4103751

[B50] May W. J. Henderson F. Gruber R. B. Discala J. F. Young A. P. Bates J. N. (2013b). Morphine has latent deleterious effects on the ventilatory responses to a hypoxic-hypercapnic challenge. Open J. Mol. Integr. Physiol. 3, 134–145. 10.4236/ojmip.2013.33019 25045592 PMC4103749

[B51] Montandon G. Ren J. Victoria N. C. Liu H. Wickman K. Greer J. J. (2016). G-protein-gated inwardly rectifying potassium channels modulate respiratory depression by opioids. Anesthesiology 124, 641–650. 10.1097/ALN.0000000000000984 26675532 PMC4755838

[B52] Mori T. Shibasaki Y. Matsumoto K. Shibasaki M. Hasegawa M. Wang E. (2013). Mechanisms that underlie μ-opioid receptor agonist-induced constipation: differential involvement of μ-opioid receptor sites and responsible regions. J. Pharmacol. Exp. Ther. 347, 91–99. 10.1124/jpet.113.204313 23902939

[B53] Moss R. B. Pryor M. M. Baillie R. Kudrycki K. Friedrich C. Reed M. (2020). Higher naloxone dosing in a quantitative systems pharmacology model that predicts naloxone-fentanyl competition at the opioid mu receptor level. PLoS One 15, e0234683. 10.1371/journal.pone.0234683 32544184 PMC7297366

[B54] Neumueller S. E. Buiter N. Hilbert G. Grams K. Taylor R. Desalvo J. (2023). Effects of sub-lethal doses of fentanyl on vital physiologic functions and withdrawal-like behaviors in adult goats. Front. Physiol. 14, 1277601. 10.3389/fphys.2023.1277601 37885800 PMC10598602

[B55] Peat S. J. Hanna M. H. Woodham M. Knibb A. A. Ponte J. (1991). Morphine-6-glucuronide: effects on ventilation in normal volunteers. Pain 45, 101–104. 10.1016/0304-3959(91)90170-3 1907362

[B56] Rossi G. C. Bodnar R. J. (2021). Interactive mechanisms of supraspinal sites of opioid analgesic action: a festschrift to dr. Gavril W. Pasternak. Gavril W. Pasternak. Cell Mol. Neurobiol. 41, 863–897. 10.1007/s10571-020-00961-9 PMC11448623 32970288

[B57] Scholz J. Steinfath M. Schulz M. (1996). Clinical pharmacokinetics of alfentanil, fentanyl and sufentanil. An update. Clin. Pharmacokinet. 31, 275–292. 10.2165/00003088-199631040-00004 8896944

[B58] Seckler J. M. Getsy P. M. May W. J. Gaston B. Baby S. M. Lewis T. H. J. (2023). Hypoxia releases S-nitrosocysteine from carotid body glomus cells-relevance to expression of the hypoxic ventilatory response. Front. Pharmacol. 14, 1250154. 10.3389/fphar.2023.1250154 37886129 PMC10598756

[B59] Seckler J. M. Grossfield A. May W. J. Getsy P. M. Lewis S. J. (2022). Nitrosyl factors play a vital role in the ventilatory depressant effects of fentanyl in unanesthetized rats. Biomed. Pharmacother. 146, 112571. 10.1016/j.biopha.2021.112571 34953397 PMC8776621

[B60] Shang Y. Filizola M. (2015). Opioid receptors: structural and mechanistic insights into pharmacology and signaling. Eur. J. Pharmacol. 763, 206–213. 10.1016/j.ejphar.2015.05.012 25981301 PMC4584181

[B61] Suzuki J. El-Haddad S. (2017). A review: fentanyl and non-pharmaceutical fentanyls. Drug Alcohol Depend. 171, 107–116. 10.1016/j.drugalcdep.2016.11.033 28068563

[B62] Torralva R. Eshleman A. J. Swanson T. L. Schmachtenberg J. L. Schutzer W. E. Bloom S. H. (2020). Fentanyl but not morphine interacts with nonopioid recombinant human neurotransmitter receptors and transporters. J. Pharmacol. Exp. Ther. 374, 376–391. 10.1124/jpet.120.265561 32513839 PMC7430447

[B63] Trescot A. M. Datta S. Lee M. Hansen H. (2008). Opioid pharmacology. Pain Physician 11, S133–S153. 10.36076/ppj.2008/11/s133 18443637

[B64] Tschirhart J. N. Li W. Guo J. Zhang S. (2019). Blockade of the human ether A-go-go-related gene (hERG) potassium channel by fentanyl. Mol. Pharmacol. 95, 386–397. 10.1124/mol.118.114751 30665971

[B65] Tschirhart J. N. Zhang S. (2020). Fentanyl-induced block of hERG channels is exacerbated by hypoxia, hypokalemia, alkalosis, and the presence of hERG1b. Mol. Pharmacol. 98, 508–517. 10.1124/mol.119.119271 32321735

[B66] Tyler B. M. Guarnieri M. (2023). Long-acting opioid analgesics for acute pain: pharmacokinetic evidence reviewed. Vet. Sci. 10, 372. 10.3390/vetsci10060372 37368758 PMC10302878

[B67] Vadivelu N. Mitra S. Hines R. L. (2011). Peripheral opioid receptor agonists for analgesia: a comprehensive review. J. Opioid Manag. 7, 55–68. 10.5055/jom.2011.0049 21434585

[B68] Valentino R. J. Volkow N. D. (2018). Untangling the complexity of opioid receptor function. Neuropsychopharmacology 43, 2514–2520. 10.1038/s41386-018-0225-3 30250308 PMC6224460

[B69] van der Schier R. Roozekrans M. van Velzen M. Dahan A. Niesters M. (2014). Opioid-induced respiratory depression: reversal by non-opioid drugs. F1000Prime Rep. 6, 79. 10.12703/P6-79 25343036 PMC4173639

[B70] Vo Q. N. Mahinthichaichan P. Shen J. Ellis C. R. (2021). How μ-opioid receptor recognizes fentanyl. Nat. Commun. 12, 984. 10.1038/s41467-021-21262-9 33579956 PMC7881245

[B71] Yamamoto A. Sugimoto Y. (2010). Involvement of peripheral mu opioid receptors in scratching behavior in mice. Eur. J. Pharmacol. 649, 336–341. 10.1016/j.ejphar.2010.07.039 20863827

