# Supplementary material for: Fentanyl activates opposing opioid and non-opioid receptor systems that control breathing
Source: Front Pharmacol. 2024 Apr 18;15:1381073. doi: 10.3389/fphar.2024.1381073 (PMC11063261; doi:10.3389/fphar.2024.1381073)
Supplement: Supplementary file 1 [file DataSheet1.docx]

**Supplementary File**

**Supplementary Table S1**

Definition of ventilatory parameters described in this study

| **Parameter** | **Abbreviation** | | **Units** | | **Definition** |
| --- | --- | --- | --- | --- | --- |
| **A. Directly recorded parameters** | | | | | |
| Frequency of breathing | | Freq | | breaths/min | Rate of breathing |
| Inspiratory Time | | Ti | | sec | Duration of inspiration |
| Expiratory Time | | Te | | sec | Duration of expiration |
| End Inspiratory Pause | | EIP | | msec | Pause between end of inspiration start of expiration |
| End Expiratory Pause | | EEP | | msec | Pause between end of expiration and start of inspiration |
| Relaxation time | | RT | | sec | Decay of expiration to 36% maximum |
| Tidal Volume | | TV | | ml | Volume of inspired air per breath |
| Peak Inspiratory Flow | | PIF | | ml/sec | Maximum inspiratory flow |
| Peak Expiratory Flow | | PEF | | ml/sec | Maximum expiratory flow |
| Expiratory flow at 50% | | EF_50_ | | ml/sec | Expiratory flow at 50% expired TV |
| Non-eupneic breathing index | | NEBI | | % | % of non-eupneic breaths per epoch |
| **B. Derived parameters** | | | | | |
| Minute Ventilation | | MV = Freq x TV | | ml/min | Total volume of air inspired per min |
| Ti/Te | | none | | none | Inspiratory quotient |
| PEF/PIF | | none | | none | Flow balance |
| Expiratory Delay | | Te-RT | | No units | Difference in lengths of Te and RT |
| Inspiratory Drive | | TV/Ti | | ml/sec | Central urge to inhale |
| Expiratory Drive | | TV/Te | | ml/sec | Central urge to exhale |
| NEBI/Frequency | | NEBI/Freq | | %/(breath/min) | Balanced rejection index |

**Supplementary Figure S1**

**Supplementary Figure S1.** Relationships between peak inspiratory flow (PIF), peak expiratory flow (PEF), relaxation time (RT) and expiratory time (Te).

**Supplementary Figure S2**

**A.**

**B.**

**C.**

**Supplementary Figure S2.** **Panels A, B and C:** Cumulative responses (%change from Pre-values) in recorded and calculated parameters over the 15-min period following injection of fentanyl (75 μg/kg, IV) in male rats that received subsequent injections of vehicle or NLXmi (1.0 or 2.5 mg/kg, IV). The data are presented as mean ± SEM. There were 4 rats in each group. **p* < 0.05, significant change from Pre-values. There were no between-group differences for any parameter (*p* > 0.05, for all comparisons). **ANOVA statistics:** Freq: F_2,9_ = 0.05, *p* = 0.952; TV: F_2,9_ = 1.63, *p* =0.249; MV: F_2,9_ = 0.32, *p* =0.737; Ti: F_2,9_ = 0.53, *p* = 0.608; Te: F_2,9_ = 0.25, *p* = 0.785; Ti/Te: F_2,9_ = 0.01, *p* = 0.993; EIP: F_2,9_ = 1.96, *p* = 0.20; EEP: F_2,9_ = 1.04, *p* = 0.394; PIF: F_2,9_ = 1.17, *p* = 0.353; PEF: F_2,9_ = 0.06, *p* = 0.944; PIF/PEF: F_2,9_ = 1.46, *p* = 0.282; EF_50_: F_2,9_ = 0.01, *p* = 0.987; RT: F_2,9_ = .30, *p* = 0.749; Te-RT: F_2,9_ = 0.36, *p* = 0.710; InspD: F_2,9_ = 1.04, *p* =0.393; ExpD: F_2,9_ = 1.24, *p* =0.334; NEBI: F_2,9_ = 1.99, *p* = 0.193; NEBI/F: F_2,9_ = 2.27, *p* = 0.0.160.

**Supplementary Figure S3**

**A. Total cumulative fentanyl responses in female rats prior to administration of vehicle or NLXmi**

**B. Total cumulative responses at 15-min post-fentanyl in female rats**

**Supplementary Figure S3. Panel A:** Cumulative responses (%change from Pre-values) recorded over the 15-min period after injection of fentanyl (75 μg/kg, IV) in female rats that received subsequent injections of vehicle or NLXmi (1.0 or 2.5 mg/kg, IV). The data are presented as mean ± SEM. There were 4 rats in each group. **p* < 0.05, significant change from Pre-values. There were no between-group differences for any parameter (*p* > 0.05, for all comparisons). **ANOVA statistics:** Freq: F_2,9_ = 0.28, *p* = 0.765; TV: F_2,9_ = 2.19, *p* =0.168; MV: F_2,9_ = 1.10, *p* =0.373. **Panel B:** Cumulative responses (%change from Pre-values) at 15-min post-fentanyl in the above groups of female rats. The data are presented as mean ± SEM. There were 4 rats in each group. There were no significant differences from Pre-values in any group and no between-group differences for any parameter (*p* > 0.05, for all comparisons). **ANOVA statistics:** Freq: F_2,9_ = 0.16, *p* = 0.858; TV: F_2,9_ = 1.45, *p* =0.285; MV: F_2,9_ = 0.84, *p* =0.838.
